# A New Medical Dictionary

**Published:** 1890-11

**Authors:** 


### Book Notices.

A New Medical Dictionary—Including all the words and phrases used in medicine, with their proper pronunciation and definitions. Based on recent medical literature. By Geo. M. Gould, B. A., M. D., Ophthalmic Surgeon to the Philadelphia Hospital and Clinical Chief of Ophthalmological Department, German Hospital, Philadelphia. Pp. 519; 1890. P. Blackiston, Son & Co.

This is a very handy, practical dictionary. It not only defines and properly pronounces medical words and phrases, but it contains elaborate tables of the bacilli, micrococci, leucomaïnes, ptomaïnes, etc., of the arteries, ganglia, muscles, nerves, and plexuses; of weights and measures, thermometers, etc.; and appendices, containing classified tables with analyses of the waters of the mineral springs of the United States and tables of vital statistics. B.

